# Use of multivitamins, folic acid and herbal supplements among breast cancer survivors: the black women's health study

**DOI:** 10.1186/1472-6882-11-30

**Published:** 2011-04-15

**Authors:** Mireille Bright-Gbebry, Kepher H Makambi, JoyAnn Phillips Rohan, Adana A Llanos, Lynn Rosenberg, Julie R Palmer, Lucile L Adams-Campbell

**Affiliations:** 1 Lombardi Comprehensive Cancer Center, Georgetown University, 3970 Reservoir Road, NW, Research Building, E501, Washington, DC 20057, USA; 2 Slone Epidemiology Center at Boston University, 1010 Commonwealth Ave, Boston, MA 02215, USA

## Abstract

**Background:**

Complementary and alternative medicine (CAM) use, including herbals and multivitamin supplements, is quite common in the U.S., and has been shown to be highest in breast cancer survivors. However, limited data are currently available for CAM usage among African Americans. Thus, we sought to determine the prevalence of multivitamins, folic acid and herbal supplement usage in African American breast cancer survivors, and to compare the characteristics of users and nonusers.

**Methods:**

A cohort study of breast cancer survivors, who completed the 1999 Black Women's Health Study questionnaire and self-reported having been diagnosed with breast cancer between 1995 and 1999, comprised the study population. In this study, the intake of natural herbs, multivitamins and folic acid at least three days per week within the past two years was used as a proxy for typical usage of this complimentary alternative medicine (CAM) modality.

**Results:**

A total of 998 breast cancer survivors were identified. Overall, 68.2% had used either herbals or multivitamin supplements or both. The three most frequently used herbals were garlic (21.2%), gingko (12.0%), and echinacea (9.4%). The multivariate analysis determined that single marital status (OR = 1.58; 95%CI: 1.04-2.41), and alcohol consumption of 1-3 drinks per week (OR = 1.86, 95%CI: 1.28-2.68) were significantly associated with increased herbal use. Multivitamin use was significantly lower among obese women (OR = 0.66, 95%CI: 0.46-0.94) and current smokers (OR = 0.53, 95%CI: 0.34-0.82).

**Conclusions:**

A significant number of African American breast cancer survivors are using herbals and multivitamins as CAM modality. Additional research is needed to understand the impact of herbals and multivitamins in African American breast cancer survivors.

## Background

Complementary and alternative medicine (CAM), including herbals and multivitamin supplements, is widely used in the U.S. general population [1-6]. Among U.S. adults, it was found that 18% surveyed in 2002 consumed herbals according to evidence-based indications [6]. Herbals (natural products), including echinacea (19.8%) and ginseng (14.1%) [5,7] are two of the most commonly used. Women use more CAM (69%) overall compared to men [8,9] and CAM use has been particularly common among women 30-69 years old, who engage in positive health behaviors (e.g. non-smoking,) and those with pre-existing health conditions [7,10-12]. However, limited data are available on CAM usage among African Americans. In a survey of U.S. households conducted from 1998-2004, Kelly et al., [13] found that the prevalence of use of any herbal/natural product during the past week was lowest in African Americans (9.5%) and highest in whites (19%). NHANES III and NHIS data also show a lower prevalence of herbal supplement use or vitamins in African Americans [14,15].

CAM usage has been reported to be high among adult cancer survivors, [16-18] with estimates ranging from 36% to 84% [18,19]. CAM use is highest among breast cancer survivors as compared to survivors of other tumor sites [20]. Herbals are commonly used in breast cancer patients in particular [21]. Brown et al., [22] demonstrated that African Americans use herbals for disease prevention, health promotion and treatment of disease conditions but are less likely to disclose their herbal use to their health-care providers than Caucasians [23].

There are limited data available on the use of CAM and more specifically herbals, multivitamins, and folic acid, which we will refer to as CAM in this study, among African American breast cancer survivors. The aims of this study were to determine the prevalence of CAM use in African American breast cancer survivors and to determine the factors associated with CAM use.

## Methods

### Study design

The present study includes a cohort study of breast cancer survivors in the Black Women's Health Study (BWHS). The BWHS is a national prospective cohort of African American women in the United States [24]. In 1995, 59,000 women between the ages of 21-69 years enrolled in the study by responding to mailed questionnaires targeting African American women audiences including Essence magazine subscribers, members of several African American professional associations, and relatives and friends of early respondents. The baseline questionnaire collected information on demographics, medical history and medication usage, and lifestyle factors including diet, cigarettes smoking and alcohol intake. Since the inception of the study, follow-up questionnaires have been mailed to participants every 2 years to update information on exposures and to identify new or recurrent illnesses such as breast cancer. Follow-up rates have been over 80% for each questionnaire cycle since 1995. The Institutional Review Boards of Boston University Medical Center and Georgetown University approved the study protocol.

### Study population

The study population comprised women who reported a diagnosis of breast cancer before enrollment in the study in 1995 or during the first four years of follow-up through 1999 and who completed the CAM use section, i.e., herbals, multivitamins, and folic acid of the 1999 questionnaire. A total of 998 breast cancer survivors were included in the present study. Breast cancer cases were identified through responses on biennial follow-up questionnaires. Pathology data were obtained through participant medical records and through state cancer registries. Medical records and cancer registry data were also used to confirm self-reported cases of breast cancer.

### Independent variables

The current data were obtained from the 1999 questionnaire which asked about herbal and multivitamin use over a two-year period from March 1997 to March 1999. The herbals listed on the survey were echinacea, garlic, ginger, St. John's wort, ginkgo, chamomile, feverfew, hawthorn, milk thistle, goldenseal, ginseng, aloe, Ephedra products, and cat's claw. Participants marked all herbals that applied. Separate questions asked about multivitamins and/or folic acid usage. Breast cancer survivors were classified as users if they used any herbals, multivitamins or folic acid at least three days per week during the previous 2-year period. Socio-Demographic characteristics were self-reported and included age (continuous), education, height, weight, marital status (married/living as married, single) and family history of breast cancer (in a mother or sibling).

Body Mass Index (BMI) *(*kg/m^2^*) *was calculated as weight/height^2 ^and categorized into three groups; ≤25 (normal), 25-29 (overweight) and ≥30 (obese). Cigarette Smoking was dichotomized as non-smokers and current smokers and current alcohol consumption was defined as the number of drinks consumed per week and grouped as non-drinker, 1-3 drinks/week and ≥ 4 drinks/week. We categorized physical activity within the past year into two clusters: walking for exercise (e.g., housework, bowling, gardening), and vigorous physical activity (e.g., basketball, swimming, aerobics); and dichotomized as ≤ 2 hour/week or >2 hour/week of vigorous physical activity. Participants were classified as either pre- or postmenopausal. Hormone Replacement Therapy (HRT) was ascertained by a dichotomous variable answering the question, "*Between March 1997 and March 1999, have you taken female hormones for menopause*?"

### Statistical analysis

The distribution of socio-demographic and other characteristics was presented as percentages, and comparisons of these characteristics between herbals and multivitamins/folic acid users and non-users were done using chi-square tests and t-tests for categorical and continuous variables, respectively. Prevalence odds ratios and the corresponding 95% confidence intervals from unconditional logistic regressions were used to determine the factors associated with the use of herbals or multivitamins. SAS version 9.2 was used to conduct these analyses and the level of significance was set at 0.05.

## Results

Among the 998 breast cancer survivors, 681 (68.2%) had used either herbals or vitamin supplements or both; 53.6% of the women had used vitamin supplements, and 39% had used one or more herbal supplements. The three most frequently used herbals were garlic (21.2%), gingko (12.0%), and echinacea (9.4%), (Table 1). Separately, we conducted analyses to determine whether there were differences in CAM usage by years since diagnosis (<5 years since diagnosis versus ≥5 years since diagnosis). Mean ages of the breast cancer survivors were 54.7 and 52.1 years for users and non-users, respectively. CAM use was significantly higher among survivors who were well educated, postmenopausal and non-smokers (Table 2). However, we found no significant differences for use of garlic, gingko, echinacea, or any other herbal, vitamin, or combination by years since diagnosis (chi square p-values >0.05) (data not shown).

**Table 1:** Herbals and vitamins use among breast cancer survivors, BWHS, 1999

	Reporting use (%) (n = 998)
**Herbals**	
**Any herbal**	39.0
Garlic	21.2
Gingko	12.0
Echinacea	9.4
Ginseng	8.0
Goldenseal	5.1
Ginger	4.7
Chamomille	4.6
Aloe	4.3
St. John's Wort	4.1
Milk Thisle	2.0
Hawthorn	1.0
Cat's Claw	0.9
Feverfew	0.4
Ephedra products	0.3

**Vitamins/Minerals**	
Multivitamins	53.6
Folic-acid (alone)	4.6
**Any Herbals/Vitamins**	
One	35.6
Two	16.7
Three	6.7
At least four	8.3

**Table 2:** Demographic and other characteristics of breast cancer survivors by herbals and multivitamins/folic acid use, BWHS

Variable	CAM USERS (n = 681)	CAM NON-USERS (n = 317)
*Age, years (Mean ± SD)*	54.7 ± 9.5	52.1 ± 9.4
Education, years (%)*		
≤12	20.7	28.1
13-15	28.5	29.3
≥ 16	50.7	42.6
*BMI, kg/m*^*2 *^*(Mean ± SD)*	29.3 ± 6.0	30.5 ± 9.4
*BMI, kg/m*^*2 *^*(%)*		
<25	23.2	19.6
25-29	37.0	33.1
≥ 30	38.0	42.7
*Married (%)*		
Married/living as married	40.1	41.3
Single	14.4	12.3
Divorced/separated/widowed	42.6	41.0
Menopausal Status (%)*		
Premenopausal	12.3	16.7
Postmenopausal	71.1	59.0
*Hormonal replacement therapy use (%)*		
No	89.0	86.8
Yes	9.7	8.8
*Family history of breast cancer (%)*		
No	81.1	77.9
Yes	18.9	22.1
Cigarette smoking (%)*		
Non-smoker	90.8	83.2
Current smoker	9.2	16.8
*Alcohol consumption (%)*		
Non-drinker	70.2	73.8
1-3 drinks/week	16.7	12.9
≥ drinks/week	8.5	6.6
*Walking for exercise (%)*		
≤ 2 hours/week	80.0	84.9
>2 hours/week	12.6	9.5
*Vigorous physical activity (%)*		
≤ 2 hours/week	81.8	88.0
>2 hours/week	9.1	6.0

*p value <0.05, percentages do not add to 100% due to missing values

Multivariate analysis of selected variables and herbals and multivitamins/folic acid use among breast cancer survivors are presented in Table 3. The variables that were significantly associated with increased herbal use in African American breast cancer survivors were marital status/single relative to married/living as married (OR = 1.58; 95% CI:1.04-2.41, and alcohol consumption of 1-3 drinks per week compared to non-drinkers (OR = 1.86, 95% CI:1.28-2.68). Single marital status was associated with increased multivitamin use (OR = 1.56, 95%CI: 1.02-2.39). Multivitamin use was significantly lower among obese women (OR = 0.66, 95%CI: 0.46-0.94) and current smokers (OR = 0.53, 95%CI: 0.34-0.82).

**Table 3:** Multivariate analysis of selected variables associated with use of herbals and multivitamins/folic acid among breast cancer survivors

Variable	HERBALS USERS	MULTIVITAMINS/ FOLIC ACID
	**Adjusted OR**^**+ **^**(95% CI)**	**Adjusted OR**^**+ **^**(95% CI)**
*Age (years)*	1.01 (0.99,1.03)	1.02 (1.01,1.04)*
*Education*		
≤ 12	1.00	1.00
13-15	0.96 (0.66,1.40)	1.38 (0.95,1.99)
≥ 16	0.95 (0.67,1.39)	1.29 (0.91,1.81)
*BMI (kg/m*^*2*^*)*		
<25	1.00	1.00
25-29	0.97 (0.67,1.39)	0.84 (0.58,1.20)
≥ 30	0.75 (0.34,1.63)	0.66 (0.46,0.94)*
*Marital status*		
Married/living as married	1.00	1.00
Single	1.58 (1.04,2.41)*	1.56 (1.02,2.39)*
Divorced/separated/widowed	1.16 (0.86,1.56)	1.05 (0.78,1.40)
*Menopausal Status*		
Premenopausal	1.00	1.00
Postmenopausal	1.37 (0.86,2.20)	1.16 (0.74,1.36)
*Hormonal replacement therapy use*		
No	1.00	1.00
Yes	1.00 (0.63,1.57)	0.86 (0.55,1.36)
*Family history of breast cancer*		
No	1.00	1.00
Yes	0.73 (0.52,1.03)	0.94 (0.68,1.31)
*Cigarette smoking*		
Non-smoker	1.00	1.00
Current smoker	0.62 (0.39,0.97)*	0.53 (0.34,0.82)*
*Alcohol consumption*		
Non-drinker	1.00	1.00
1-3 drinks/week	1.86 (1.28,2.68)*	1.30 (0.89,1.88)
≥ 4 drinks/week	1.50 (0.94,2.39)	1.37 (0.85,2.20)
*Walking for exercise*		
≤ 2 hours/week	1.00	1.00
>2 hours/week	1.43 (0.94,2.17)	1.28 (0.84,1.94)
*Vigorous physical activity*		
≤ 2 hours/week	1.00	1.00
>2 hours/week	1.37 (0.83,2.23)	1.20 (0.72,1.98)

*Significant odds ratios, p < 0.05; each variable is adjusted for the other variables

## Discussion

To our knowledge, this is the largest study of CAM usage, as defined by herbals and multivitamins use, among African American breast cancer survivors. Several national cross-sectional and case-control studies have reported on the use of complementary and alternative medicine in breast cancer survivors [2,16,19,25], but few have reported on herbals and multivitamins [13,14,22,26], and in African American breast cancer survivors in particular [13,27,28].

Our study indicates that over 50% of breast cancer survivors use at least one type of CAM, including herbs or multivitamins, consistent with other cohort studies of breast cancer survivors [25,29]. In this investigation, herbals are by far the most popular CAM products used compared to multivitamins. Excepted for garlic, which is the number one herbal used in our research, gingko, echinacea, and ginseng are among the top three herbs used, consistent with other breast cancer survivor studies [16,21,25]. A key finding in this research is that the prevalence rate among African American breast cancer survivors is comparable to that observed in other predominantly white cohorts of breast cancer survivors [19,25]. Our results were also similar to reports of CAM use including larger samples of African Americans [5,7,13].

Of note, our multivariate analysis indicated that both obesity (BMI ≥ 30) and smoking were significantly associated with lower use of herbs and multivitamins among African American breast cancer survivors in our cohort, consistent with other cohort studies [30], and U.S. national samples [15,26,31], indicating a correlation of herbal and multivitamin use with healthy behaviors.

The findings of this study add to the current research on one of the most commonly used CAM modalities, herbals/multivitamins, among breast cancer survivors and particularly in an ethnic-specific group of African American women. A limitation of the study is the lack of data on reasons for using CAM; herbals may be used for both disease treatment and disease prevention. Further research is warranted to examine reasons for CAM use and specifically herbals/multivitamins prior to and after diagnosis. Additionally, questions on other modalities of CAM (e.g., home remedies, prayer) relevant for this population would be of interest.

## Conclusions

Complementary and alternative medicine including herbals and multivitamin use is widespread within the U.S. population and among persons with chronic illnesses such as cancer. Previously reported differences among racial/ethnic minorities prompted us to examine the prevalence of CAM usage in African American breast cancer survivors. Further research is needed to understand the impact of CAM use and specifically herbals and multivitamins in African American breast cancer survivors. Future analyses of these data may help explain some of the observed variation in the use of individual CAM therapies within African Americans.

## Competing interests

The authors declare that they have no competing interests.

## Authors' contributions

MBG interpreted data and took the lead on drafting the manuscript. KHM performed all statistical analysis. JPR and AAL participated in the writing and editing of the manuscript. LR and JP made substantial contribution to acquisition of all data, and revised and reviewed the version to be published. LAC conceived the study, coordinated it, and critically reviewed it for intellectual content. All authors read and approved the final manuscript.

## Pre-publication history

The pre-publication history for this paper can be accessed here:

http://www.biomedcentral.com/1472-6882/11/30/prepub

## References

[B1] Matthews AK Sellergren SA Huo D List M Fleming G Complementary and alternative medicine use among breast cancer survivors J Altern Complement Med 2007 13 555 562 10.1089/acm.2007.03-9040 17604560

[B2] Tindle HA Davis RB Phillips RS Eisenberg DM Trends in use of complementary and alternative medicine by US adults: 1997-2002 Altern Ther Health Med 2005 11 42 49 15712765

[B3] Gardiner P Kemper KJ Legedza A Phillips RS Factors associated with herb and dietary supplement use by young adults in the United States BMC Complement Altern Med 2007 7 39 10.1186/1472-6882-7-39 18053129 PMC2213683

[B4] Arcury TA Suerken CK Grzywacz JG Bell RA Lang W Quandt SA Complementary and alternative medicine use among older adults: ethnic variation Ethn Dis 2006 16 723 731 16937611

[B5] Hsiao AF Wong MD Goldstein MS Yu HJ Andersen RM Brown ER Becerra LM Wenger NS Variation in complementary and alternative medicine (CAM) use across racial/ethnic groups and the development of ethnic-specific measures of CAM use J Altern Complement Med 2006 12 281 290 10.1089/acm.2006.12.281 16646727

[B6] Bardia A Nisly NL Zimmerman MB Gryzlak BM Wallace RB Use of herbs among adults based on evidence-based indications: findings from the National Health Interview Survey Mayo Clin Proc 2007 82 561 566 10.4065/82.5.561 17493422 PMC1964882

[B7] Barnes PM Bloom B Nahin RL Complementary and Alternative Medicine Use Among Adults and Children: United States, 2007 2010 National Center for Complementary and Alternative Medicine, National Institutes of Health 19361005

[B8] Mueller CM Mai PL Bucher J Peters JA Loud JT Greene MH Complementary and alternative medicine use among women at increased genetic risk of breast and ovarian cancer BMC Complement Altern Med 2008 8 17 10.1186/1472-6882-8-17 18447953 PMC2391138

[B9] Upchurch DM Chyu L Greendale GA Complementary and Alternative Medicine Use Amont American Women: Findings from the National Health Interview Survey, 2002 Journal of Women's Health 2007 16 102 113 10.1089/jwh.2006.M074 17324101

[B10] Nahin RL Dahlhamer JM Taylor BL Barnes PM Stussman BJ Simile CM Blackman MR Chesney MA Jackson M Miller H Health behaviors and risk factors in those who use complementary and alternative medicine BMC Public Health 2007 7 217 10.1186/1471-2458-7-217 17723149 PMC2031902

[B11] Ni H Simile C Hardy AM Utilization of complementary and alternative medicine by United States adults: results from the 1999 national health interview survey Med Care 2002 40 353 358 10.1097/00005650-200204000-00011 12021691

[B12] Barnes PM Powell-Griner E McFann K Nahin RL Complementary and alternative medicine use among adults: United States, 2002 Adv Data 2004 1 19 15188733

[B13] Kelly JP Kaufman DW Kelley K Rosenberg L Mitchell AA Use of herbal/natural supplements according to racial/ethnic group J Altern Complement Med 2006 12 555 561 10.1089/acm.2006.12.555 16884347

[B14] Fennell D Determinants of supplement usage Prev Med 2004 39 932 939 10.1016/j.ypmed.2004.03.031 15475026

[B15] Radimer KL Subar AF Thompson FE Nonvitamin, nonmineral dietary supplements: issues and findings from NHANES III J Am Diet Assoc 2000 100 447 454 10.1016/S0002-8223(00)00137-1 10767902

[B16] Carpenter CL Ganz PA Bernstein L Complementary and alternative therapies among very long-term breast cancer survivors Breast Cancer Res Treat 2009 116 387 396 10.1007/s10549-008-0158-3 18712472 PMC3101874

[B17] Velanovich V Hallal N Shah M Patterns of usage of complementary and alternative medicine in general surgical patients Int J Surg 2006 4 206 211 10.1016/j.ijsu.2006.03.005 17462352

[B18] Morris KT Johnson N Homer L Walts D A comparison of complementary therapy use between breast cancer patients and patients with other primary tumor sites Am J Surg 2000 179 407 411 10.1016/S0002-9610(00)00358-5 10930491

[B19] Buettner C Kroenke CH Phillips RS Davis RB Eisenberg DM Holmes MD Correlates of use of different types of complementary and alternative medicine by breast cancer survivors in the nurses' health study Breast Cancer Res Treat 2006 100 219 227 10.1007/s10549-006-9239-3 16821087

[B20] Velicer CM Ulrich CM Vitamin and mineral supplement use among US adults after cancer diagnosis: a systematic review J Clin Oncol 2008 26 665 673 10.1200/JCO.2007.13.5905 18235127

[B21] Graham RE Ahn AC Davis RB O'Connor BB Eisenberg DM Phillips RS Use of complementary and alternative medical therapies among racial and ethnic minority adults: results from the 2002 National Health Interview Survey J Natl Med Assoc 2005 97 535 545 15868773 PMC2568705

[B22] Brown CM Barner JC Richards KM Bohman TM Patterns of complementary and alternative medicine use in African Americans J Altern Complement Med 2007 13 751 758 10.1089/acm.2006.6392 17931068

[B23] Kuo GM Hawley ST Weiss LT Balkrishnan R Volk RJ Factors associated with herbal use among urban multiethnic primary care patients: a cross-sectional survey BMC Complement Altern Med 2004 4 18 10.1186/1472-6882-4-18 15575960 PMC539258

[B24] Palmer JR Boggs DA Adams-Campbell LL Rosenberg L Family history of cancer and risk of breast cancer in the Black Women's Health Study Cancer Causes Control 2009 20 1733 1737 10.1007/s10552-009-9425-9 19760028 PMC2794688

[B25] Greenlee H Kwan ML Ergas IJ Sherman KJ Krathwohl SE Bonnell C Lee MM Kushi LH Complementary and alternative therapy use before and after breast cancer diagnosis: the Pathways Study Breast Cancer Res Treat 2009 117 653 665 10.1007/s10549-009-0315-3 19184414 PMC2991094

[B26] Kennedy J Herb and supplement use in the US adult population Clin Ther 2005 27 1847 1858 10.1016/j.clinthera.2005.11.004 16368456

[B27] Lee MM Lin SS Wrensch MR Adler SR Eisenberg D Alternative therapies used by women with breast cancer in four ethnic populations J Natl Cancer Inst 2000 92 42 47 10.1093/jnci/92.1.42 10620632

[B28] Kronenberg F Cushman LF Wade CM Kalmuss D Chao MT Race/ethnicity and women's use of complementary and alternative medicine in the United States: results of a national survey Am J Public Health 2006 96 1236 1242 10.2105/AJPH.2004.047688 16735632 PMC1483863

[B29] Caan B Sternfeld B Gunderson E Coates A Quesenberry C Slattery ML Life After Cancer Epidemiology (LACE) Study: a cohort of early stage breast cancer survivors (United States) Cancer Causes Control 2005 16 545 556 10.1007/s10552-004-8340-3 15986109

[B30] Gunther S Patterson RE Kristal AR Stratton KL White E Demographic and health-related correlates of herbal and specialty supplement use J Am Diet Assoc 2004 104 27 34 10.1016/j.jada.2003.10.009 14702580

[B31] Bertisch SM Wee CC McCarthy EP Use of complementary and alternative therapies by overweight and obese adults Obesity (Silver Spring) 2008 16 1610 1615 10.1038/oby.2008.239 18451783 PMC2776037

